# Valsalva-Like Retinopathy Secondary to Pancytopenia following Induction of Etoposide and Ifosfamide

**DOI:** 10.1155/2015/985303

**Published:** 2015-01-28

**Authors:** Robert A. Prinzi, Steven Saraf, Ankit Desai, Oscar Kuruvilla, Uday R. Desai

**Affiliations:** ^1^Department of Ophthalmology, Henry Ford Hospital, 2799 W. Grand Boulevard, Detroit, MI 48202, USA; ^2^Vanderbilt Eye Institute, 2311 Pierce Avenue, Nashville, TN 37232, USA

## Abstract

Etoposide and ifosfamide are chemotherapeutic agents used frequently in the treatment of sarcomas and hematologic malignancies. Ocular side effects are rarely reported. We describe a case of a patient on etoposide and ifosfamide who presented with unilateral vision loss, anemia, and thrombocytopenia. The patient was found to have a large subinternal limiting membrane hemorrhage in the right eye that is thought to be related to his anemia and thrombocytopenia. The hemorrhage resolved spontaneously after 10 days. This case illustrates how bone marrow suppression by chemotherapeutic agents may indirectly contribute to retinal hemorrhages resulting in at least transient vision loss.

## 1. Introduction

Sub-ILM hemorrhages have been reported in cases of Valsalva retinopathy, Terson's syndrome, blood dyscrasias, and blunt facial trauma. Usually, these hemorrhages are induced by mechanical injury or pressure gradients associated with a central process, such as in Terson's syndrome [1].

Spontaneous sub-ILM hemorrhages are rarely reported. A case described by de Maeyer et al. describes a patient with acute myeloid leukemia with severe thrombocytopenia who developed a sub-ILM hemorrhage. The case required vitrectomy and excision of the hemorrhagic cyst resulting in 20/50 vision. The chemotherapy regimen in this case was not reported [1]. Another case described by Borges et al. describes a patient with non-Hodgkin's lymphoma on chemotherapy who presented with hand-motions vision in both eyes. The right eye had a large preretinal hemorrhage that was treated promptly with vitrectomy and membrane peel. The patient recovered vision to 20/50. The other eye was operated a week later but did not achieve similar visual recovery. The authors suggested that early intervention may be beneficial in such cases [2].

Medical management may be an appropriate course in cases of sub-ILM hemorrhages. Sub-ILM hemorrhages have been reported in cases of blood dyscrasias such as idiopathic thrombocytopenic purpura (ITP) or aplastic anemia. These cases were also spontaneous and not secondary to trauma or Valsalva mechanism. In a reported case of ITP, vitrectomy was offered to resolve a subhyaloid and vitreous hemorrhage, but the patient declined intervention. The underlying condition of ITP was treated medically and the hemorrhages improved with recovery of vision [3]. Mansour et al. reported a series of 37 patients with aplastic anemia who experienced retinal hemorrhages, some of which were sub-ILM. The patients were treated medically with red blood cell and platelet transfusions and followed by the ophthalmology service. The authors discuss various treatment options undertaken by the ophthalmology service including observation and Nd:YAG membranotomy. However, the authors do not report the outcomes of each modality [4]. Although Nd:YAG membranotomy spares the patient the more invasive option of vitrectomy, it may not be possible if nonclearing vitreous hemorrhage precludes an adequate view. Vitrectomy with membrane peel has been shown to be effective in managing sub-ILM hemorrhages and may be preferred in such cases [1].

## 2. Case Report

A 19-year-old male with a history of metastatic Ewing's sarcoma presented with vision loss in the right eye. He endorsed headache but denied ophthalmalgia, pain with eye movements, recent vomiting, or other Valsalva-inducing activities.

**Figure 1:**
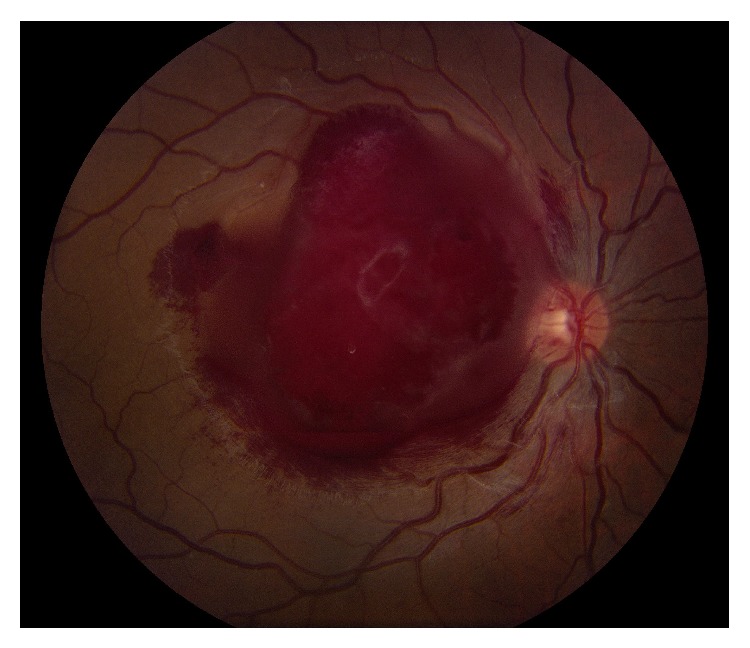
Color fundus photo of the right eye on the day of presentation. This shows the large preretinal hemorrhage involving almost the entire macula.

**Figure 2:**
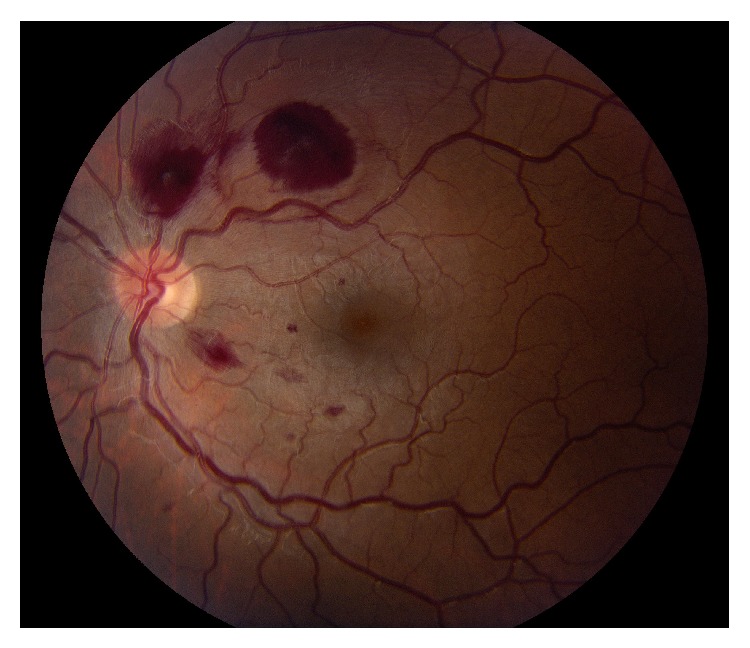
Color fundus photo of the left eye on the day of presentation. Note the large intraretinal and flame shaped hemorrhages superior to the optic disc.

His visual acuity was 20/400 in the right eye and 20/20 in the left eye. A relative afferent pupillary defect was present in the right eye with normal pupillary response in the left eye. Confrontational visual fields, extraocular movements, and intraocular pressures were all within normal limits. Dilated fundus exam of the right eye revealed a large preretinal hemorrhage encompassing a large area within the macula (Figure 1). Dot-blot and flame hemorrhages were noted along the arcades in the left eye sparing the fovea (Figure 2). Patches of myelinated nerve fibers were noted in both eyes. The patient was unable to see the color plates with his right eye due to central blurring. Color vision was normal in the left eye. Initial laboratory work-up was significant for hemoglobin of 4.9 g/dL and platelet count of 17 K/*μ*L with a normal INR. Computerized tomography (CT) scan of the brain did not reveal any intracranial hemorrhages or other acute processes.

**Figure 3:**
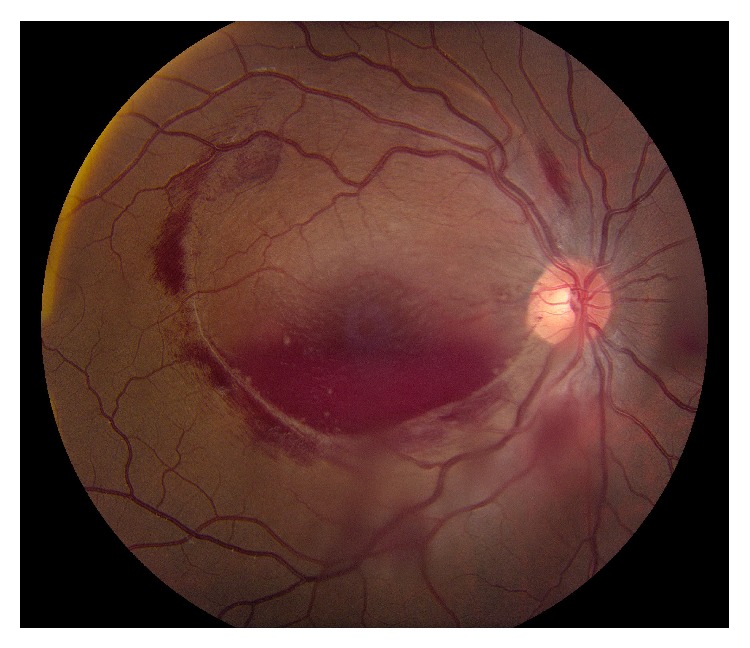
Color fundus photo of the right eye 10 days after presentation. The preretinal hemorrhage has spontaneously drained in the vitreous.

**Figure 4:**
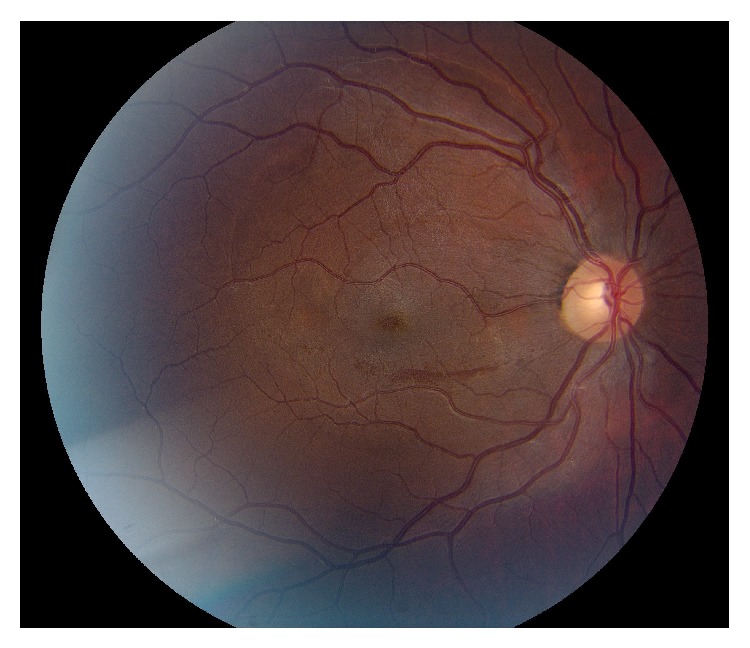
Color fundus photo of the right eye 3 months after presentation. Retinal hemorrhage has almost completely resolved.

After treatment with packed red blood cells and platelets, his hemoglobin improved to 10.3 g/dL and platelet count improved to 78 K/*μ*L. The patient was observed closely without aggressive surgical intervention given his systemic status. Ten days after presentation, the sub-ILM hemorrhage in the right eye was noted to have spontaneously drained into the vitreous, with only a minimal boat-shaped hemorrhage remaining inferiorly (Figure 3). This coincided with his improvement in vision to count fingers. Three months after presentation the hemorrhage in the right eye resolved and his vision improved to 20/20 with resolution of his relative afferent pupillary defect (Figure 4).

**Figure 5:**
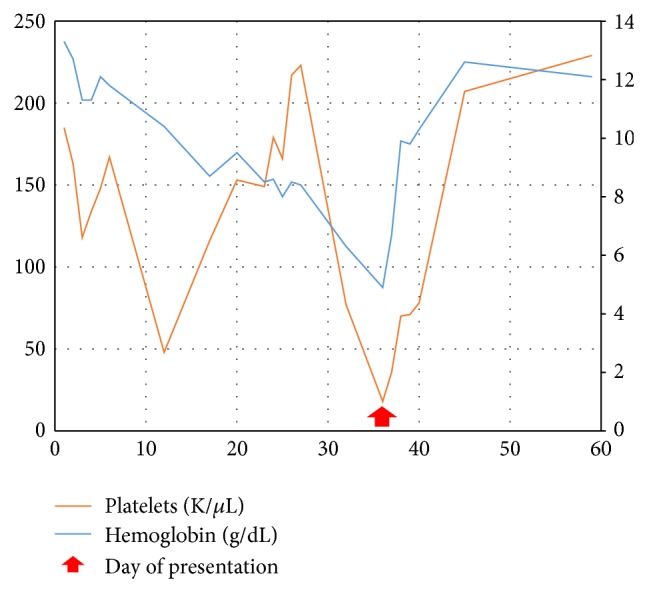
The patient's hemoglobin and platelets are graphed in the days after initiating etoposide and ifosfamide. The minimum value of both parameters occurs on the day of presentation when retinal hemorrhage was diagnosed.

The patient had undergone two cycles of chemotherapy with ifosfamide and etoposide prior to presentation and was noted to be pancytopenic during his initial course of therapy with minimum values of hemoglobin and platelets occurring on the day of presentation (Figure 5). He was continued on ifosfamide and etoposide and was given prophylactic transfusions as needed prior to his chemotherapeutic infusions. There were no further ocular complications or hemorrhages for the duration of his treatment.

## 3. Discussion

Low platelet levels, high MCV, and anemia have been demonstrated to correlate with the prevalence of retinal hemorrhages using univariate regression analysis [5]. Prior studies have also shown that the prevalence of retinopathy in patients with anemia or thrombocytopenia is 28.3%. However, when both conditions are present in the same patient, the prevalence increases to 42%, suggesting a synergistic effect. The critical value for anemia appears to be a hemoglobin level under 8 g/dL, at which level approximately 70% of patients were found to have fundus lesions [5, 6]. The pathophysiologic mechanisms behind anemia and thrombocytopenia-related retinopathy explain their synergistic effects. In anemia, decreased oxygen carrying capacity results in damage to the endothelial capillary cell lining of retinal vessels [7]. Normally, platelets provide a degree of protection for the compromised endothelial cell lining. However, in concomitant thrombocytopenia, there are insufficient platelets to adequately protect the endothelial lining, placing the tissue at risk for hemorrhage [6].

Our patient became symptomatic at the nadir of his platelet and hemoglobin counts. The patient's hemoglobin fluctuated throughout the course of treatment, secondary to blood transfusions, while his platelet count steadily declined. This finding supports a need to monitor and treat any associated retinopathy in cooperation with Hematology-Oncology to prevent recurrence of hemorrhage and maximize the chance of recovery after hemorrhage has occurred.

Etoposide is a topoisomerase inhibitor that interferes with the unwinding of DNA during replication [8]. Ifosfamide is an alkylating agent that adds an alkyl group to DNA, preventing replication enzymes from adequately accessing the template strand [9]. Both agents strongly affect neoplastic cells due to their tendency to proliferate more rapidly than normal cells. The major systemic toxicity associated with etoposide is bone marrow suppression [10]. Ifosfamide has also been documented to cause bone marrow suppression in addition to hemorrhagic cystitis, nephrotoxicity, and neurotoxicity [11]. In regard to ocular side effects, intra-arterial etoposide has been documented to cause arterial thrombosis with central retinal artery occlusion being one possible corollary [12]. Cisplatin and etoposide when used in conjunction have been reported to lead to symptomatic retinopathy with abnormal electroretinography (ERG) and visual evoked response (VER), though this was thought to be more attributed to the platinum component of cisplatin [13]. Ifosfamide has been reported to lead to blurring of vision, though the cause was not described. It is also known to cause florid conjunctivitis [14].

To our knowledge, the current case is the only report of etoposide and ifosfamide associated with anemia and thrombocytopenia resulting in spontaneous sub-ILM hemorrhage. As in the previously cited cases, we considered treatment with vitrectomy versus Nd:YAG membranotomy [15]. Delaying intervention runs the risk of scar formation in the pocket of hemorrhage and secondary vision loss. The mechanism of scarring is thought to be due to retinal pigment epithelial cell migration to the site of hemorrhage with the development of scar tissue similar to epiretinal membrane formation seen in proliferative vitreoretinopathy [16]. However, immediate vitrectomy was delayed in this patient due to the degree of his thrombocytopenia and anemia. Fortunately, his hemorrhage drained spontaneously after only ten days following platelet and packed red blood cell transfusions. In this case, close observation did not result in visual morbidity with the final visual acuity recovering to 20/20.

We report a case related to pancytopenia that resulted in a good outcome with observation only. This highlights the importance of treating the underlying mechanism of anemia-induced retinopathy with close observation while maintaining a low threshold to intervene when necessary.
